# Effects of Adolescents’ Lifestyle Habits and Body Composition on Bone Mineral Density

**DOI:** 10.3390/ijerph18116170

**Published:** 2021-06-07

**Authors:** Chang-Sook Han, Hyo-Kyung Kim, Suhee Kim

**Affiliations:** 1 Division of Nursing, Graduate School, Hallym University, 1 Hallymdaehak-gil, Chuncheon-si 135-841, Gangwon-do, Korea; hcs414@gmail.com (C.-S.H.); noproblem15@naver.com (H.-K.K.); 2 School of Nursing and Research Institute of Nursing Science, Hallym University, 1 Hallymdaehak-gil, Chuncheon-si 135-841, Gangwon-do, Korea

**Keywords:** adolescents, body composition, bone mineral density, lifestyle habits

## Abstract

The incidence of osteoporosis is increasing as the population ages, as is the need to manage and prevent it. Adolescence is the period when the fastest development of bone mass takes place. Increasing adolescents’ maximum bone mass and avoiding the risk factors for its loss are effective for preventing osteoporosis. This study investigated the factors influencing adolescents’ bone mineral density (BMD). The participants were 126 middle- and high-school students from Gangwon-do; 47.6% (*n* = 60) were male, with an average age of 15 (range 12–18) years of age. It was found that age, carbonated beverages, snacks, and calcium supplements were variables that showed significant differences in adolescents’ BMD. Additionally, through correlation analysis, it was found that height, weight, body mass index (BMI), body water, protein, minerals, body fat mass, and skeletal muscle mass were correlated with BMD. Multiple regression analysis identified age, calcium supplements, BMI, body fat mass, and skeletal muscle mass as BMD-associated factors. These results show that adolescents’ BMD is higher with lower body fat mass, higher BMI and skeletal muscle mass, and a higher intake of calcium supplements.

## 1. Introduction

With the population aging worldwide, the incidence of osteoporosis is increasing, and the disease has been recognized as a serious public health problem [1]. Osteoporosis due to low bone mass is a problem faced globally by women aged over 50 [2]. In South Korea, among adults aged over 50, 22.4% have osteoporosis, 47.9% have osteopenia, and almost 80% have lower than normal bone density [3]. An increase in osteoporosis cases due to aging has been suggested as an important public health problem, with its management and prevention being sought at the national level [4]. Osteoporosis, a representative metabolic disease caused by decreased bone mineral density (BMD), is not a problem by itself, but it can seriously impact the quality of life of the elderly through increased functional dependence and risk of fractures [5]. The loss of mobility and independence following a fracture not only lead to decreased quality of life in old age [6] but also cause a rapid increase in medical and social expenses [7]. Therefore, preventive measures are necessary to manage osteoporosis, with the most effective method being to accumulate bone mass and subsequently delay its loss [8]. Adolescence is a growth period in which rapid increases and accumulation of bone mass are observed. BMD increases after the fusion of the growth plates in this period, leading to a significant increase in bone mass at the end of puberty [9]. Therefore, increasing peoples’ maximum bone mass during adolescence to avoid risk factors associated with its loss later in life would be the most effective way to prevent future osteoporosis.

Genetic factors, such as sex and race—which cannot be modified [10,11]—diseases, such as genetic diseases, endocrine diseases, and complications due to chronic diseases [12], and certain lifestyle habits, such as physical activity and diet, affect adolescents’ BMD [11]. Studies have shown that increased physical activity has beneficial effects on BMD [1,13]. For example, it was observed that BMD in adults increases following greater amounts of physical activity during their growth phase [14]. Additionally, regular physical activity during adolescence improves peoples’ body composition, cardiorespiratory fitness, and overall bone health. In particular, weight-bearing and resistance exercises, which are affected by gravity, have been reported to show positive effects on peoples’ bone density [15]. Dietary habits are another major factor influencing one’s BMD. Calcium and vitamin D intake have both been shown to have positive effects on female hormones, such as estrogen and calcitonin, thereby increasing BMD [16]. Moreover, calcium has been shown to decrease peoples’ risk of vertebral compression fractures [17]. However, Korea’s national health statistics of 2013 indicate that the average daily calcium intake of Korean adolescents is only 56.7% of the recommended amount, with an insufficient calcium intake rate being observed in 81.5% of this population, which suggests a seriously inadequate calcium consumption [18].

Furthermore, societal preference and interest in having a skinny body often causes an unbalanced nutritional intake, resulting in people being underweight during adolescence, thus negatively affecting their BMD [19]. Many previous studies have demonstrated that anthropometric measurements, including height, weight, and body mass index (BMI), are correlated with bone density [13,20,21]. However, other studies have suggested that excessive increases in weight and body fat during adolescence have negative effects on bone-density formation [22].

Studies have reported different results on the factors affecting BMD, and, to date, research related to BMD has mostly been conducted on adult women [11], menopausal women [21], and college students [16]. However, adolescence is a known critical period for the foundation of adults’ health. It is therefore necessary to analyze the factors affecting bone mass in multiple dimensions and, in particular, to assess the relationship between BMD and peoples’ lifestyles and overall body composition. This study examined the effects of adolescents’ lifestyle habits and body composition on their BMD and can provide basic data for the prevention of future osteoporosis and improvement of their overall bone health. The study’s hypotheses are as follows: (1) adolescents’ BMD will vary according to their lifestyle; (2) adolescents’ BMD will vary according to their body composition; and (3) lifestyle habits and body composition will affect adolescents’ BMD.

## 2. Materials and Methods

### 2.1. Research Design and Data Collection

This was a secondary data analysis based on previously collected data, with review exemption being approved by the Institutional Review Board of the researcher’s institution (IRB approval no.: HIRB-2020-EX001).

**Figure 1:**
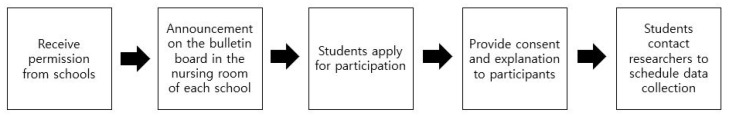
Flowchart of the participants’ recruitment.

The primary survey data were collected to investigate factors affecting scoliosis in adolescents from 8 January to 21 February 2019. Data were collected from the students after receiving permission from the principals and nurses of randomly selected middle and high schools in C city, Gangwon-do. Notices to recruit participants were displayed on the bulletin board of the nursing room in each school. Students were informed about the study’s purpose as well as the confidentiality of the collected data. Additionally, the survey methods and body measurements (including those on bone density and body composition) were explained. Written explanations and consent forms for this study were provided by the school nurse to the students who wished to participate. Once the students, who had voluntarily agreed to participate, contacted the researcher, they were provided with specific dates for the collection of their anthropometric measurements in the researcher’s institution (Figure 1). To guarantee participants’ rights and privacy, the researcher explained that participation in this study was voluntary, and that they could withdraw at any time without any negative consequences. Additionally, the researcher explained that the collected information would be used for research purposes only, stored in a safe place, and accessible only to the researcher.

Data were collected at a designated location in the researcher’s institution. Approximately 30 min were spent collecting each participant’s body measurements (BMD and body composition) and completing the questionnaire. Once the data were collected, each participant was compensated for their participation, which included transportation expenses and a gift card. Data were collected by two researchers who were familiar with the SONOST 3000 manufacturer’s manual and inspection protocol and four trained investigators. The researchers taught the investigators how to test twice before starting the study. During the study, data collection was conducted at the same location and using the same tool by the researchers and trained investigators.

### 2.2. Research Subjects

The target population included middle- or high-school students from Gangwon-do, with the accessible population being students from selected middle and high schools. Among the adolescents who agreed to participate in the study and whose guardians also consented, only those without chronic conditions, such as thyroid diseases, heart diseases, diabetes, or cancer, were included in the study. Moreover, athletes with a high muscle mass and those with diseases, such as growth hormone deficiency, that may have affected their weight were excluded from the study. The number of required subjects was determined using the G*Power program. In the multiple regression analysis with a middle-size effect of 0.15, a significance level (*p*) of 0.05, power (1-β) of 0.80, and nine predictor variables, a minimum of 114 subjects was required. Considering a 10% drop-out rate, 126 subjects were selected for this study, all of whom were included in the final analysis without any dropouts. 

### 2.3. Research Tools

In this study, the questionnaire items utilized mainly assessed the subjects’ general characteristics, such as their age, sex, and lifestyle habits (including their diet and physical activities) as well as their physical measurements, including their height, weight, body composition, and BMD.

#### 2.3.1. BMD 

Bone mineral density was measured using a quantitative ultrasound (QUS) method via a mobile BMD meter (SONOST 3000, Korea), with the average bone quality index (BQI) being assessed at the right and left calcaneus. The SONOST 3000 measures broadband ultrasound attenuation and the speed of sound, which are both related to temperature, and presents BQI values that are adjusted for any temperature error. For more accurate measurements, the SONOST 3000 was calibrated once daily using Phantom, in accordance with the manufacturer’s instructions.

While dual-energy X-ray absorptiometry (DXA) is recommended for diagnosing osteoporosis, the use of QUS is increasing due to problems related to exposing adolescents to radiation. Additionally, encouraging results were achieved in a previous study after adjusting for factors such as age, gender, and pubertal status [23]. The QUS method was used in this study because it is cheaper than the DXA method, there is no exposure to radiation, and because it yielded good results in previous studies. Thus, since the QUS method was used rather than DXA; the bone density in the calcaneus and not the lumbar spine or femur was measured. 

In addition, BMD measured by DXA is expressed in g/cm^2^ (areal bone density); however, the values are converted into T-scores or Z-scores and reported. The T-score shows the difference in standard deviation compared to the mean value of a young group of the same sex. The Z-scores show the difference, in terms of standard deviation, between the mean values of those in same age group of the same sex. Using T- and Z-scores was deemed inappropriate for this study because the participants were South Korean adolescents, and there is a lack of data on Z-scores. Therefore, the BQI value, obtained using the SONOST 3000 machine, was analyzed as a variable for bone mineral density.

#### 2.3.2. Body Composition

A body composition analyzer (Inbody 3.0 Biospace, Seoul, Korea) was used to measure the adolescents’ weight, body water, protein levels, mineral levels, body fat mass, and skeletal muscle mass, which was done while the subjects were standing upright, without shoes, on the machine. Their height was measured using a German-made extensometer (seca213). Finally, height and weight were then entered into a body composition analyzer (Inbody 3.0 Biospace, Korea) to calculate subjects’ BMI.

To increase the reliability of the body composition measurements, participants were asked to avoid vigorous exercise 24 h prior to data collection. The measurements were taken after urination and defecation to remove as much body residue as possible from the participants. Moreover, since the body’s composition may change based on its temperature, the temperature of the room where the body composition analysis was performed was maintained at 20–25 degrees Celsius.

#### 2.3.3. Dietary Habits

Items surveyed in previous studies [11,16] as well as items from the questionnaires for the Korea Youth Risk Behavior Web-based Survey (KYRBS) [24] were used as references to assess the study subjects’ dietary habits. 

The KYRBS annually surveys about 60,000 youth health behaviors across the country and uses them to plan national youth health-promotion projects [24]. Regarding dietary habits, the survey respondents were asked, “How many days in the last 7 days did you eat breakfast?” They were asked to choose between 0 and 7 days. In addition, the survey asked, “How often have you eaten fruits, soda, fast food, ramen, snacks, vegetables, milk, etc., in the last 7 days?” The respondents were then asked to choose one of the following options: not eaten in the last 7 days/ 1–2 times a week/ 3–4 times a week/ 5–6 times a week/ once a day/ twice a day/ at least 3 times a day.

In this study, we asked the participants how often they consumed protein-based foods, vegetables, fruits, milk and dairy products, carbonated drinks, fried foods, snacks, instant noodles, and coffee. They were then asked to choose one of the following options: not eaten in the last 7 days/ 1–2 times a week/ 3–4 times a week/ 5–6 times a week/ once a day/ twice a day/ at least 3 times a day. In this study, the proportion of those who consumed the surveyed foods every day was small; therefore, the focus was on how often they ate such foods every week.

Furthermore, the questions asked in this study about breakfast were similar to those in the KYRBS. Additionally, we asked questions regarding their intake of calcium, vitamin D, and red ginseng supplements.

#### 2.3.4. Physical Activity

The amount of physical activity per participant was evaluated using the International Physical Activity Questionnaire (IPAQ). This tool comprises seven items covering four domains and is freely downloadable from its website (https://sites.google.com/site/theipaq/home) (accessed on 1 January 2019). Using the IPAQ score protocol [25], the questionnaire classifies physical activity into three categories (high-active, moderate-active, and low-active groups).

The low-active group is the lowest level of the physical activity groups and includes people who do not fall into other categories or are not active. The moderate-active group included those who met any of the following three criteria: (1) three or more days of vigorous activity for at least 20 min per day; (2) five or more days of moderate-intensity activity or walking for at least 30 min per day; or (3) five or more days of any combination of walking, moderate-intensity, or vigorous intensity activities, achieving at least 600 MET-min/week. The high-active group comprised those who met any of the following two criteria: (1) vigorous-intensity activity on at least three days and accumulating at least 1500 MET-minutes/week, or (2) seven or more days of any combination of walking, moderate-intensity, or vigorous intensity activities, achieving a minimum of at least 3000 MET-minutes/week.

### 2.4. Data Analysis

SPSS IBM 25.0 was used for all statistical analyses. A frequency analysis was performed on the adolescents’ general characteristics, their lifestyle habits, and their body composition to obtain both frequencies and percentages for each variable. Additionally, a descriptive statistical analysis was performed to calculate the mean and standard deviation of each variable. The Shapiro–Wilk test was performed to assess the normality of BQI according to each variable’s category. All variables were normally distributed. Any differences in BMD according to each participant’s general characteristics and lifestyle habits were analyzed via a *t*-test and an ANOVA. A Pearson’s correlation analysis was performed to assess the relationship between each participant’s body composition and their BMD, with a multiple regression analysis being conducted to evaluate the variables’ effects on the participating adolescents’ BMD. 

All the results were considered to be statistically significant when *p* < 0.05.

## 3. Results

### 3.1. General and Lifestyle-Related Characteristics of the Study Subjects

In total, 47.6% (*n* = 60) and 52.4% (*n* = 66) of the subjects in this study were male and female, respectively, and their ages ranged from 12 to 18, or an average of 15.

Regarding the subjects’ weekly dietary habits, 33.3% (*n* = 42), 34.9% (*n* = 44), 25.4% (*n* = 32), and 33.3% (*n* = 42) had more than five intakes of protein-based food, vegetables, fruits, and milk and dairy products, respectively. Additionally, 23% (*n* = 29) of the subjects consumed fatty foods, such as fast food, fries, and pork belly, more than five times per week, while 14.3% (*n* = 18) consumed snacks more than five times weekly. In total, 35.7% and 13.5% of the subjects consumed instant noodles and coffee more than three times per week, respectively. Fifty percent (*n* = 63) of the participating adolescents ate breakfast less than three times weekly, while the other half ate it more than four times a week. Moreover, 7.1% (*n* = 9), 7.9% (*n* = 10), and 6.3% (*n* = 8) of the subjects consumed, on a weekly basis, calcium supplements, red ginseng, and vitamin D supplements, respectively. The proportion of those who underwent moderate levels of physical exercise was the highest at 32.5% (*n* = 41), with those who engaged in mild and high levels of exercise coming to 23.8% (*n* = 30) and 15.9% (*n* = 20), respectively. A total of 26.2% (*n* = 33) of the adolescents spent less than five hours a day in a sitting position, with 20.6% (*n* = 26) and 18.3% (*n* = 23) spending five to nine hours and more than 10 h per day, respectively, in a sitting position. A total of 57.1% (*n* = 72) of the subjects reported more than seven hours of sleep a day, while the remaining 42.9% (*n* = 54) slept less than seven hours daily. Additionally, the proportion of the subjects who spent less than 40 min engaging in outdoor activities was 64.3% (*n* = 81) (Table 1).

### 3.2. Differences in BMD According to Subjects’ General Characteristics and Lifestyle Habits

Differences in the participating adolescents’ BMD were observed according to their age (t = −2.679, *p* = 0.008), consumption rate of carbonated drinks (t = 2.099, *p* = 0.038), consumption of snacks (t = 2.293, *p* = 0.024), and consumption of calcium supplements (t = −2.032, *p* = 0.044). In contrast, no differences in BMD according to sex as well as the intake of protein, vegetables, fruits, milk and dairy products, coffee, fried foods, instant noodles, breakfast, red ginseng, vitamin D supplements, and subjects’ physical activity levels and sleep time, were observed (Table 1).

### 3.3. Body Composition Distribution

The mean relevant body composition measurements of the subjects were as follows: height, body weight, body water, protein levels, mineral levels, body fat mass, skeletal muscle mass, and body fat percentage were 164.4 cm, 58.2 kg, 30.9%, 8.2%, 3.03%, 15.9 kg, 23 kg, and 26.8%, respectively.

The minimum, maximum, and mean sample group BQI were 54.25, 111.05, and 81.46 (standard deviation (SD) = 12.68), respectively. The mean BMI of the participants was 21.3 kg/m^2^ (SD = 3.57), with a minimum value of 14.8 kg/m^2^ and a maximum of 35.4 kg/m^2^ (Table 2).

Among boys, the average BQI of the 12–14-year group was 79.23, and the average BQI of the 15–18-year group was 87.76. Among girls, the average BQI of the 12–14-year group was 76.74, and the average BQI of the 15–18-year group was 81.66 (Appendix A). Please refer to Table A1 in the Appendix A for specific body-composition values with respect to gender and age.

### 3.4. Correlation between Body Composition and BMD

The analysis of the correlation between the participants’ body composition and their BMD revealed that their height (r = 0.275, *p* = 0.002), weight (r = 0.336, *p* = 0.000), BMI (r = 0.283, *p* = 0.001), body water (r = 0.356, *p* = 0.000), protein levels (r = 0.365, *p* = 0.000), mineral levels (r = 0.364, *p* = 0.000), body fat mass (r = 0.177, *p* = 0.048), and skeletal muscle mass (r = 0.363, *p* = 0.000) were all positively correlated with their BMD (Table 3).

As a result of analyzing the correlation by separating sex and age, BQI had a statistically significant positive correlation with body weight, body water, and body fat mass in both genders aged 15–18 (Table A2).

### 3.5. Factors Affecting BMD

A multiple regression analysis was performed to identify the factors affecting adolescent BMD. Their age and intake of carbonated drinks, snacks, and calcium supplements, all of which resulted in significant differences in BMD according to the univariate analysis, as well as body composition factors (including height, weight, BMI, body water, protein levels, mineral levels, body fat mass, and skeletal muscle mass) were included in the linear regression analysis using the Enter method. Weight, which was found to be highly correlated with other body components, was excluded from the analysis. Among all factors, those with a variance inflation factor (VIF) higher than 10 were excluded. The other factors had a VIF ranging between 1.029 and 7.794 (Table 4). When body fat mass and skeletal muscle mass were entered together, their multicollinearity exceeded 10. Thus, they were entered separately into Models 1 and 2.

Model 1 included the participants’ body fat mass, with the multiple regression analysis showing that their age (β = 0.206, *p* = 0.015), calcium supplement intake (β = 0.190, *p* = 0.020), BMI (β = 0.576, *p* = 0.002), and body fat mass (β = −0.390, *p* = 0.032) were significant factors affecting their BMD. The total explanatory power of the regression model (F = 6.046, *p* = 0.000) is 26.4%. In Model 2, the participants’ skeletal muscle mass was entered. The multiple regression analysis revealed that their age (β = 0.216, *p* = 0.011), calcium supplementary intake (β = 0.191, *p* = 0.018), and skeletal muscle mass (β = 0.504, *p* = 0.024) were significant factors affecting their overall BMD. The total explanatory power of the developed regression model (F = 6.149, *p* = 0.000) was 26.7%. In other words, these results suggest that relatively high BMI, skeletal muscle mass, and calcium supplement intake, in addition with lower body fat mass, lead to overall increased BMD (Table 4). Please refer to Table A3 in the Appendix A for multiple regression including sex.

## 4. Discussion

This study aimed to assess the effects of lifestyle habits and body composition on BMD during adolescence, when bone growth is the most active, with the goal of promoting bone health in adolescents to prevent future osteoporosis. BMD during adolescence is highly associated with future fracture risk during adulthood [26]. The results reveal that BMD during adolescence is higher at 15–18 years than it is at 12–14 years. In other words, BMD increases with rise in age, skeletal muscle mass, and calcium supplement intake.

In general, BMD gradually increases in children and adolescents with age and then decreases after they reach peak bone mass. Specifically, BMD increases as puberty progresses [27]. In a previous study on healthy children and adolescents aged 6 to 14, BMD was also found to increase with age [28]. Another study reported BMD increasing with age among female middle-school students [29], which is consistent with the findings of this study.

From our univariate analysis on the relationship between adolescents’ body composition and their BMD, we found that numerous variables, including the participants’ height, weight, BMI, body water, protein levels, mineral levels, body fat mass, and skeletal muscle mass, were related to their overall BMD. In the multiple regression analysis, variables that were highly related to each other were excluded due to their multicollinearity. As a result, it was observed that only BMI, body fat mass, and skeletal muscle mass significantly affect adolescents’ BMD.

Many previous studies have reported that anthropometric measurements, such as peoples’ height, weight, and BMI, are positively correlated with their overall BMD [13,20,21]. Moreover, in one systematic review that analyzed 27 studies comparing BMD of normal and overweight adolescents, it was found that BMD was significantly higher in those who were overweight [17,30]. Conversely, a study that analyzed the results of the United States’ National Health and Nutrition Examination Survey on adolescents, excluding those who were underweight, showed that total body areal bone mineral density (aBMD) and lumbar spine aBMD decreased as fat mass increased in both male and female adolescents [31]. In addition, pelvic aBMD has been found to decrease as total fat mass increases in male adolescents, which was the opposite of what was observed in female adolescents. Therefore, it has been verified that the effects of weight on bone density differ depending on the individual’s sex and body parts [31]. Another study found that in both male and female participants, more lean mass led to positive effects on their BMD; however, in female adolescents who lacked lean mass, their body fat was found to have a statistically significant positive effect on their overall bone density [32]. Furthermore, some studies have shown that excessive body fat in adolescents negatively affects their bone mass [33]. In contrast, body fat was found to have positive effects on bone mass in relatively underweight adolescents [34]. In Model 1 of the current study, both BMI and body fat were included as variables, and we observed that BMD increases with decreased body fat and increased BMI. In general, this specific finding is consistent with the results of previous studies. However, in future studies, the differential effects of sex need to be assessed, and the various bone densities of different body parts must be measured. The effects of being overweight versus being underweight need to be further investigated to assess the relationship between body fat mass and BMD.

In Model 2, body fat was excluded, with BMI and skeletal muscle mass added as variables while considering their multicollinearity, to assess the relationship between body composition and BMD. In Model 1, BMI was a factor that significantly increased BMD; however, it was not a significant factor in Model 2. This finding suggests that skeletal muscle mass, rather than BMI, has a greater effect on adolescents’ BMD. In one study of Korean female middle-school students, which developed an estimation formula for BMD, it was found that age, skeletal muscle mass, and BMI significantly affected their BMD, and the greatest effect was observed from skeletal muscle mass [29]. These findings are consistent with those of the current study. A study conducted on adults aged 20–49 reported a positive correlation between skeletal muscle mass and BMD. Additionally, low skeletal muscle mass was identified as a risk factor for low BMD in premenopausal women; however, no significant effects were observed in men [35]. In other words, this past study reported that the effects of skeletal muscle mass differ depending on the degree of BMD in both male and female subjects. Therefore, studies using methods such as a quantile regression analysis by separating the sexes and distinguishing cases of low versus high BMD are necessary to assess these variables’ effects on BMD more accurately.

Furthermore, our study assessed the effects of lifestyle habits on BMD and found that calcium supplement intake has positive effects on adolescents’ BMD. Calcium is known to increase overall BMD and reduce the risk of vertebral fractures [16,17]. One study that analyzed the relationship between the intake of calcium, milk, and vitamin D and BMD in Spanish adolescents found that both calcium and milk were positively associated with BMD in male adolescents, but only vitamin D was positively correlated with BMD in female adolescents [36]. Moreover, in Chinese adolescents, an increase in BMD and lean mass due to calcium supplement intake at various doses was observed for over two years. In that study, an intake of 230 mg or more of calcium supplements per day was found to increase participants’ total body bone-mineral content and lean mass in male adolescents; however, no effects were observed in female adolescents, even when they consumed high doses of the provided calcium supplement [37]. Conversely, calcium supplement intake in Caucasian female adolescents has been found to significantly increase BMD after 18 months when compared to a control group that received a placebo [38]. Similar to the results of the current study, previous research has reported that BMD increases in those who consume calcium supplements when compared to those who do not. However, the effects vary depending on the person’s sex. Therefore, future studies assessing the differential effects of calcium on BMD in different sexes are necessary. Furthermore, although the increase was not significant, BMD was found to be higher in those who spent more time engaging in outdoor activities. In future studies, it is thus necessary to analyze any differences in BMD according to sex, calcium supplement intake, and time spent undertaking outdoor activities as well as variations based on peoples’ intake of vitamin D supplements (amount/frequency/period).

In this study, too, BMD was found to significantly vary according to the participants’ consumption levels of carbonated drinks and snacks in the univariate analysis. BMD was significantly lower in those adolescents who consumed instant noodles more than three times a week when compared to those who consumed it less than twice a week, with a significance level of 0.1 in the univariate analysis. Previous studies have found that carbonated soft drink consumption is related to lower BMD in female adolescents but not in male adolescents [39,40]. Therefore, future studies that focus on the differences in individuals’ BMD according to their dietary habits across different sexes are necessary. Additionally, it is recommended that intervention programs aimed at improving peoples’ BMD include education on the negative effects of carbonated drinks, snacks, and instant noodles.

Previous studies have also reported that both physical activity and dietary habits are essential [1,13,15]. Additionally, the Centers for Disease Control and Prevention (CDC) recommends that children and adolescents perform 60 min of moderate- to high-intensity exercises daily [41], as regular physical activity improves one’s cardiopulmonary capacity and strengthens bones and muscles. Additionally, the CDC guidelines propose that children and adolescents should perform physical activities that strengthen both their muscles and bones at least three days a week [41]. The current study demonstrates that BMD is higher in those who exercised well and spent less time in a sitting position. However, significant differences were not observed in the univariate analysis, perhaps because physical activity was only defined as including aerobic exercises. Aerobic exercises do have positive effects on the health of adolescents. However, weight-bearing and resistance exercises, which are greatly affected by gravity, have more significant effects on bone health [15]. Therefore, it is necessary for future studies to assess the relationship between strength training and BMD.

The study has some limitations. First, it was a secondary data analysis, and therefore, the number of variables related to the characteristics affecting BMD was limited. Smoking and drinking, despite the intake of vitamin D supplements (amount/frequency/period) and strength training, may also have significant effects on BMD. However, these variables could not be included in this study. Furthermore, though the intake of milk and dairy products were investigated, the intake of calcium from other foods was not considered. Second, some of the data, such as exercise, dietary habits, and time spent outdoors, were reported by the participants and were not observed or measured by the researcher. Hence, they may not be as accurate. Third, it was not possible to analyze each group based on sex. Therefore, it is suggested that future studies identify factors that affect BMD based on sex and age. Notwithstanding these limitations, this study is significant in that the effects of skeletal muscle mass on BMD were more clearly assessed when compared to previous research that mainly described the relationship between lean mass and BMD. Lean mass is measured by subtracting body fat from the person’s total weight and including the weight of their organs, skin, bones, water in the body, and muscle mass. Although lean mass increases with muscle mass, the latter does not necessarily increase with the former. Thus, this study is significant in that skeletal muscle mass was used to assess the factors affecting BMD. Moreover, the differences found in adolescents’ BMD according to their body composition as well as their lifestyle habits were also assessed. Therefore, this study was able to secure the basic data for the development of appropriate intervention programs to promote bone health in adolescents to prevent their developing osteoporosis in future.

## 5. Conclusions

Bone mineral density in adolescence is related to both genetic factors, such as sex and race, and environmental variables, such as diet and lifestyle habits. In particular, a person’s BMI and their lack of exercise are highly related to BMD. Adolescence is a critical period in which bone mass rapidly increases and accumulates. Thus, this study was conducted to understand the relationship between lifestyle habits and body composition, factors that affect BMD in adolescents.

We found that BMD is higher in high-school students aged 15–18, during which maximum bone mass is accumulated, when compared to those in middle school, aged 12–14. Additionally, the type of diet and amount of physical activity conducted are important contributing factors. In particular, differences in BMD were observed according to calcium supplement intake, which is known to help bone growth. Moreover, factors affecting BMD include participants’ BMI, body fat mass, and skeletal muscle mass, with BMD increasing with BMI and skeletal muscle mass. Therefore, adolescents need to aim at reducing their body fat through engaging in regular exercise and increasing their overall physical activity, while making sure to maintain a proper weight through the intake of various foods and of sufficient calcium to improve their overall BMD. 

In this study, more varied and detailed variables that affect adolescents’ BMD were not included. Therefore, it is recommended that future studies consider more diverse variables, such as sex, vitamin D supplement intake (amount/frequency/period), and strength training. The findings of this study may be used to develop physical activity and diet-education programs to promote proper bone health in adolescents, which may help prevent possible osteoporosis in adulthood or old age.

## Figures and Tables

**Table 1:** Difference of BQI (bone quality index) by characteristics of adolescents (*n* = 126).

Characteristics	Categories	*n* (%)	BQI		
			Mean ± SD	t/F	*p*
General characteristics					
Sex	Male	60 (47.6)	83.20 ± 12.91	1.482	0.141
	Female	66 (52.4)	79.87 ± 12.35		
Age	Mean ± SD	14.79 ± 1.665		−2.679	0.008
	12–14	55 (43.7)	78.21 ± 10.40		
	15–18	71 (56.3)	83.98 ± 13.74		
Diet habits					
Protein-based food (times/week)	≤4	84 (66.7)	81.95 ± 13.24	0.612	0.542
	≥5	42 (33.3)	80.48 ± 11.56		
Vegetables (times/week)	≤4	82 (65.1)	81.94 ± 12.88	0.583	0.561
	≥5	44 (34.9)	80.56 ± 12.38		
Fruits (times/week)	≤4	94 (74.6)	81.52 ± 13.21	0.095	0.925
	≥5	32 (25.4)	81.28 ± 11.18		
Milk and dairy products (times/week)	≤4	84 (66.7)	82.23 ± 12.86	0.967	0.335
	≥5	42 (33.3)	79.92 ± 12.31		
Carbonated drinks (times/week)	≤4	114 (90.5)	82.22 ± 12.62	2.099	0.038
	≥5	12 (9.5)	74.25 ± 11.31		
Fried foods (times/week)	≤4	97 (77.0)	81.86 ± 12.95	0.638	0.525
	≥5	29 (23.0)	80.14 ± 11.83		
Snacks (times/week)	≤4	108 (85.7)	82.50 ± 12.84	2.293	0.024
	≥5	18 (14.3)	75.22 ± 9.80		
Instant noodles (times/week)	≤2	81 (64.3)	82.88 ± 12.00	1.697	0.092
	≥3	45 (35.7)	79.91 ± 13.58		
Coffee (times/week)	≤2	109 (86.5)	81.37 ± 12.16	−0.197	0.844
	≥3	17 (13.5)	82.03 ± 16.05		
Number of breakfasts	≤3 days	63 (50)	82.36 ± 13.85	0.801	0.425
	≥4 days	63 (50)	80.55 ± 11.42		
Calcium supplements	No	117 (92.9)	80.83 ± 12.56	−2.032	0.044
	Yes	9 (7.1)	89.63 ± 11.97		
Red ginseng nutrients	No	116 (92.1)	81.88 ± 12.70	1.289	0.200
	Yes	10 (7.9)	76.51 ± 11.96		
Vitamin D supplements	No	118 (93.7)	81.48 ± 12.54	0.090	0.928
	Yes	8 (6.3)	81.06 ± 15.59		
Activity habits					
Physical activity	Low active	30 (23.8)	79.46 ± 13.43	1.312	0.275
	Active	41 (32.5)	82.42 ± 13.40		
	Very active	20 (15.9)	85.54 ± 11.79		
Time for sitting (hr./day)	<5	33 (26.2)	84.28 ± 11.52	0.198	0.821
	5–9	26 (20.6)	83.52 ± 14.10		
	≥10	23 (18.3)	82.03 ± 14.50		
Time for sleeping (hr./day)	<7	54 (42.9)	82.86 ± 13.25	1.076	0.284
	≥7	72 (57.1)	80.41 ± 12.22		
Outdoor activity time (min/day)	<40	81 (64.3)	80.79 ± 12.99	−0.793	0.429
	≥40	45 (35.7)	82.66 ± 12.14		

**Table 2:** Distribution of body compositions (*n* = 126).

Characteristics	Mean ± SD	Range
BQI ^a^	81.46 ± 12.68	54.25–111.05
Height (cm)	164.44 ± 8.67	148–186
Weight (Kg)	58.20 ± 13.48	38.1–118.1
BMI ^b^ (Kg/m^2^)	21.36 ± 3.57	14.8–35.4
Body water (ℓ)	30.90 ± 6.59	19.8–51.8
Protein (Kg)	8.29 ± 1.81	5.3–13.8
Minerals (Kg)	3.03 ± 0.62	2.03–5.15
Body fat mass (Kg)	15.98 ± 7.35	3.8–47.3
Skeletal muscle mass (Kg)	23.01 ± 5.45	13.9–39.9
Percent body fat (%)	26.84 ± 8.14	8.9–41.2

^a^ BQI, bone quality index; ^b^ BMI, body mass index.

**Table 3:** Correlations between body composition and BQI (*n* = 126).

	r (*p*)									
	1	2	3	4	5	6	7	8	9	10
1. BQI ^a^	1									
2. Height (cm)	0.275 ** (0.002)	1								
3. Weight (kg)	0.336 ** (0.000)	0.692 ** (0.000)	1							
4. BMI ^b^ (kg/m^2^)	0.283 ** (0.001)	0.309 ** (0.000)	0.896 ** (0.000)	1						
5. Body water (ℓ)	0.356 ** (0.000)	0.863 ** (0.000)	0.855 ** (0.000)	0.606 ** (0.000)	1					
6. Protein (kg)	0.365 ** (0.000)	0.859 ** (0.000)	0.852 ** (0.000)	0.604 ** (0.000)	0.999 ** (0.000)	1				
7. Minerals (kg)	0.364 ** (0.000)	0.862 ** (0.000)	0.905 ** (0.000)	0.669 ** (0.000)	0.982 ** (0.000)	0.980 ** (0.000)	1			
8. Body fat mass (kg)	0.177 * (0.048)	0.211 * (0.018)	0.780 ** (0.000)	0.893 ** (0.000)	0.343 ** (0.000)	0.338 ** (0.000)	0.454 ** (0.000)	1		
9. Skeletal muscle mass (kg)	0.363 ** (0.000)	0.859 ** (0.000)	0.852 ** (0.000)	0.604 ** (0.000)	1.000 ** (0.000)	1.000 ** (0.000)	0.980 ** (0.000)	0.337 ** (0.000)	1	
10. Percent body fat (%)	−0.003 (0.969)	−0.256 ** (0.004)	0.326 ** (0.000)	0.587 ** (0.000)	−0.198 * (0.026)	−0.202 * (0.024)	−0.073 (0.417)	0.830 ** (0.000)	−0.203 * (0.023)	1

* *p* < 0.05; ** *p* < 0.01; ^a^ BQI, bone quality index; ^b^ BMI, body mass index.

**Table 4:** Predictors of BQI ^a^ (*n* = 126).

	Model 1				Model 2			
	β	t	*p*	VIF ^c^	β	t	*p*	VIF
Age 15–18 (ref: 12–14)	0.206	2.459	0.015	1.123	0.216	2.569	0.011	1.141
Carbonated drinks (ref: ≤4)	−0.118	−1.442	0.152	1.078	−0.121	−1.473	0.143	1.079
Snacks (ref: ≤4)	−0.152	−1.855	0.066	1.080	−0.146	−1.777	0.078	1.084
Calcium supplements (ref: no intake)	0.190	2.367	0.020	1.029	0.191	2.391	0.018	1.029
Height (cm)	0.128	1.446	0.151	1.261	−0.220	−1.172	0.243	5.684
BMI ^b^ (kg/m^2^)	0.576	3.129	0.002	5.435	0.030	0.254	0.800	2.255
Body fat mass (kg)	−0.390	−2.165	0.032	5.198	-	-	-	-
Skeletal muscle mass (kg)	-	-	-	-	0.504	2.289	0.024	7.794
R^2^	0.264				0.267			
F	6.046				6.149			
*p*	0.000				0.000			

^a^ BQI, bone quality index; ^b^ BMI, body mass index; ^c^ VIF, variance inflation factor.

## Data Availability

The data for this study can be obtained from the corresponding author upon reasonable request.

## Appendix A

**Table A1:** Body composition classified by gender and age.

	Mean	SD	Min	Max
**Boys (12–14 years), *n* = 32**				
BQI ^a^	79.23	10.76	58.75	101.90
Height (cm)	165.37	6.96	150.50	182.60
Weight (kg)	60.61	17.09	40.5	118.1
BMI ^b^ (kg/m^2^)	21.90	4.63	14.9	35.4
Body water (ℓ)	33.36	6.22	19.8	51.8
Protein (kg)	8.93	1.66	5.3	13.8
Minerals (kg)	3.21	0.64	2.03	5.15
Body fat mass (kg)	15.12	9.95	3.8	47.3
Skeletal muscle mass (kg)	24.97	5.06	13.9	39.9
Percent body fat (%)	23.23	8.62	8.9	41.2
**Boys (15–18 years), *n* = 28**				
BQI ^a^	87.76	13.82	59.05	111.05
Height (cm)	174.37	6.28	162.00	186.00
Weight (kg)	67.68	11.63	49.4	91.0
BMI ^b^ (kg/m^2^)	22.24	3.53	17.5	30.4
Body water (ℓ)	38.40	4.13	26.8	45.6
Protein (kg)	10.38	1.15	7.2	12.4
Minerals (kg)	3.69	0.45	2.74	4.54
Body fat mass (kg)	15.22	7.76	5.2	34.7
Skeletal muscle mass (kg)	29.30	3.44	19.7	35.3
Percent body fat (%)	21.50	7.53	9.6	38.2
**Girls (12–14 years), *n*= 24**				
BQI ^a^	76.74	9.72	57.15	96.20
Height (cm)	157.22	5.19	148.00	167.70
Weight (kg)	48.85	5.67	38.6	59.2
BMI ^b^ (kg/m^2^)	19.78	2.10	15.9	24.2
Body water (ℓ)	25.03	1.95	22.1	30.0
Protein (kg)	6.66	0.55	5.8	8.1
Minerals (kg)	2.52	0.19	2.17	2.89
Body fat mass (kg)	14.63	4.13	6.5	20.7
Skeletal muscle mass (kg)	18.12	1.59	15.7	22.2
Percent body fat (%)	29.45	5.96	15.3	39.6
**Girls (15–18 years), *n* = 42**				
BQI ^a^	81.66	13.40	54.25	105.30
Height (cm)	161.23	6.52	150.20	177.90
Weight (kg)	55.39	10.03	38.1	82.7
BMI ^b^ (kg/m^2^)	21.25	3.13	14.8	29.0
Body water (ℓ)	27.40	3.75	20.7	36.1
Protein (kg)	7.33	1.01	5.6	9.6
Minerals (kg)	2.75	0.41	2.14	3.79
Body fat mass (kg)	17.91	5.91	9.3	33.2
Skeletal muscle mass (kg)	20.11	3.06	14.6	27.1
Percent body fat (%)	31.67	5.57	20.1	41.1

^a^ BQI, bone quality index; ^b^ BMI, body mass index.

**Table A2:** Correlations categorized by gender and age.

		BQI			
		Boy (12–14 years)	Boy (15–18 years)	Girls (12–14 years)	Girls (15–18 years)
		*n* = 32	*n* = 28	*n* = 24	*n* = 42
BQI ^a^	r	1	1	1	1
	*p* value				
Height	r	0.314	0.055	0.150	0.077
	*p* value	0.080	0.780	0.485	0.629
Weight	r	0.134	0.428 *	0.164	0.329 *
	*p* value	0.465	0.023	0.445	0.033
BMI ^b^	r	0.044	0.443 *	0.085	0.357 *
	*p* value	0.811	0.018	0.694	0.020
Body water	r	0.339	0.361	0.382	0.248
	*p* value	0.058	0.059	0.065	0.113
Protein	r	0.345	0.374 *	0.400	0.261
	*p* value	0.053	0.050	0.053	0.095
Minerals	r	0.294	0.352	0.360	0.283
	*p* value	0.103	0.066	0.084	0.069
Body fat mass	r	−0.059	0.375 *	−0.023	0.337 *
	*p* value	0.747	0.049	0.916	0.029
Skeletal muscle mass	r	0.348	0.377 *	0.410 8 *	0.250
	*p* value	0.051	0.048	0.046	0.111
Percent body fat	r	−0.264	0.334	−0.154	0.300
	*p* value	0.144	0.082	0.472	0.054

* *p* < 0.05; ^a^ BQI, bone quality index; ^b^ BMI, body mass index.

**Table A3:** Regression analysis including sex.

	Model 1				Model 2			
	β	t	*p*	VIF	β	t	*p*	VIF
Sex (ref: boys)	0.133	0.957	0.340	3.076	0.168	1.179	0.241	3.275
Age 15–18 (ref: 12–14)	0.170	1.848	0.067	1.351	0.175	1.929	0.056	1.336
Carbonated drinks (ref: ≤4)	−0.121	−1.469	0.144	1.079	−0.125	−1.522	0.131	1.081
Snacks (ref: ≤4)	−0.148	−1.804	0.074	1.082	−0.138	−1.676	0.097	1.092
Calcium supplements (ref: no intake)	0.185	2.299	0.023	1.033	0.185	2.320	0.022	1.033
Height (cm)	0.202	1.719	0.088	2.212	−0.292	−1.481	0.141	6.284
BMI (kg/m^2^)	0.713	3.055	0.003	8.734	−0.058	−0.413	0.680	3.148
Body fat mass (kg)	−0.538	−2.265	0.025	9.042				
Skeletal muscle mass (kg)					0.743	2.485	0.014	14.437
R2	0.270				0.276			
F	5.401				5.572			
*p*	0.000				0.000			
